# Development of Loop-Mediated Isothermal Amplification for Detection of *Leifsonia xyli* subsp. *xyli* in Sugarcane

**DOI:** 10.1155/2013/357692

**Published:** 2013-04-23

**Authors:** Jing Liu, Liping Xu, Jinlong Guo, Rukai Chen, Michael Paul Grisham, Youxiong Que

**Affiliations:** ^1^Key Lab of Sugarcane Biology and Genetic Breeding, Ministry of Agriculture, Fujian Agriculture and Forestry University, Fuzhou, Fujian 350002, China; ^2^USDA-ARS, Sugarcane Research Unit, Houma, LA 70360, USA

## Abstract

Ratoon stunt, caused by the xylem-limited coryneform bacterium *Leifsonia xyli* subsp. *xyli* (*Lxx*), is a deep bacteriosis and prevalent in most of sugarcane-producing countries. Based on loop-mediated isothermal amplification (LAMP), we developed a method for detecting *Lxx*. The major advantages of the LAMP method are visual judgment by color and time saving with only 60 min for identification of *Lxx* and without the need for costly PCR apparatus and gel scanner. In the present study, positive and negative samples detected by the LAMP method were clearly distinguishable. When total DNA extracted from internode juice was used as the template, the sensitivity of LAMP was 10 times higher than that of the conventional PCR detection. The LAMP assay is a highly specific, rapid, and sensitive method for the diagnosis of ratoon stunt caused by *Lxx* in sugarcane. This is the first report of LAMP-based assay for the detection of *Lxx* in sugarcane.

## 1. Introduction

Ratoon stunt, an important disease of sugarcane (*Saccharum *interspecific hybrids) worldwide [1], is caused by a small, fastidious, gram-positive, and xylem-limited coryneform bacterium, *Leifsonia xyli *subsp. *xyli* (*Lxx*) [2–4]. Ratoon stunt has been shown to cause up to 50% yield loss in susceptible cultivars, especially under stress such as drought condition [5]. Yield losses typically increase in the ratoon crops [6, 7]. It is reported in a previous study that during the isolation of *Lxx*, firstly, a portion of the stalk infected by *Lxx* should be washed with soap and water, rinsed with water, washed with 70 percent ethanol, and flamed. And then, an internodal section approximately 12 cm in length was aseptically excised and placed in a sterile, 50 mL conical tube for centrifugation at 1000 rpm for 1 minute to extract fibrovascular fluid where the presence of bacteria should be determined [2]. *Leifsonia xyli *subsp. *xyli* is mechanically transmitted from infected plants to healthy ones on contaminated tools and equipment, while spread from one field to another or from one geographical area to another is by infected cuttings (seed cane) [5, 8]. Control of ratoon stunt in susceptible cultivars is achieved primarily by planting seed cane free of *Lxx* and cleaning tools and equipment that may have become contaminated with infected sap from infected plants. Identifying *Lxx*-infected plants that may be the source of seed cane is difficult, because no external symptoms are produced. Internal symptoms may include a salmon pink discoloration just below the growing point of young cane and an orange-red discoloration of the nodal vascular bundles in mature cane stalks; however, these symptoms vary within and among cultivars [5]. Therefore, diagnosis of *Lxx*-infected plants is mainly by laboratory techniques.

Microscopy, serology, and DNA-based diagnostic techniques are the main methods used to detect *Lxx* [5]. Microscopy is effective in detecting *Lxx *from the internode juice but is limited by the diagnostician's ability to recognize the morphological characteristics of the bacterium and the need for the bacterium to be present at a high titer. The isolation in pure culture of *Lxx* in 1980 led to the development of immunological methods for *Lxx* [2]. However, with the use of the less specific anti-*Lxx *polyclonal antibody, the sensitivity of immunological methods is lower than polymerase chain reaction (PCR) assay [2, 9]. PCR is a relatively mature technology for detecting *Lxx*, of which the accuracy is higher than microscopic and immunological technologies. The first specific PCR primers for *Lxx* were developed in 1998, designed from the 16S–23S internal transcription spacer (ITS) ribosomal DNA of* Lxx* (NCBI accession number AF056003), and the size of the amplified fragment is 438 bp [10]. In the same year, another pair of specific PCR primers for *Lxx* was developed, and the size of the corresponding amplified fragment is 278 bp [11]. The development of a real-time PCR protocol for the detection of *Lxx* provides a more sensitive diagnostic method but requires an expensive specialized thermal cycler [12].

In 2000, Notomi et al. developed a novel DNA amplification method named loop-mediated isothermal amplification (LAMP) in which the amplification can be obtained in about 60 min with four specific primers and strand displacement DNA polymerase in isothermal conditions (approximately 65°C) eliminating the need for a thermal cycler [13]. The four specific primers include outer primers F3 and B3 and inner primers FIP and BIP, which are designed according to six regions of the target gene. Currently, LAMP is mainly applied in the fields of medicine, virus detection, food safety testing, and so forth, with less application in the detection of fungi, bacteria, nematodes in plants, and insects [14–18]. To our knowledge, LAMP has not been used in disease or pathogen detection of sugarcane.

A micropropagation sugarcane program to insure the availability of pathogen-free sugarcane seed cane was developed ten years ago in Mainland China and has been recently demonstrated in large acreage in Guangxi, Yunnan, Guangdong, and Hainan provinces and will be expanded soon.* Lxx* is the primary pathogen of concern in the production of pathogen-free sugarcane seed cane. Thus, a rapid, accurate, and low-cost detection method for *Lxx* without the need for specialized equipment is very important to support this program. The objective of this study was to develop an LAMP assay for *Lxx*.

## 2. Plant Materials and Methods 

### 2.1. Materials

The cultivar Yue Gan 18 was collected from the germplasm nursery of the Key Lab of Sugarcane Biology and Genetic Breeding, Ministry of Agriculture, China. A 439 bp specific sequence of* Lxx *located between the 16S and 23S rRNA was amplified by PCR method developed by Pan et al. [10]. In NCBI, a homology search of this sequence showed that it exists only in* Lxx* and is highly conserved. The positive plasmid (named *Lxx*-pMD18-T plasmid in this study) was the recombination of the 439 bp specific sequence and the pMD18-T vector. 

### 2.2. Primers Design

**Figure 1:**
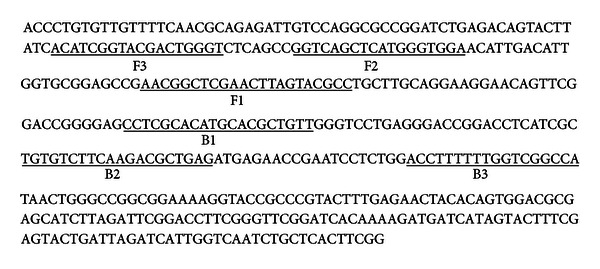
The 439 bp specific sequence of the *Lxx*. The “F1”, “F2”, “F3”, “B1”, “B2”, and “B3” are the six regions in the sequence used for the design of the LAMP primers.

Four primers, including outer primers F3 and B3 and inner primers FIP and BIP, which recognize a total of six distinct regions of the 439 bp specific sequence of *Lxx*, were designed by using the PrimerExplorer 4.0 software (http://primerexplorer.jp/e/) and synthesized by the Sangon Biotech (Shanghai) Co., Ltd. Considering that *Lxx* is only found to infect sugarcane, it is clear that these primers would not cross-react with any other bacteria that might be present on sugarcane either as pathogens or nonpathogens [2, 5–7, 10]. The purity of FIP and BIP was at HPLC grade [19]. Primer design chart and primer sequences are shown in Figure 1 and Table 1, respectively.

### 2.3. Reaction Mixture for LAMP

Initial conditions of LAMP reaction were adopted from Wang et al. [19]. It was carried out in a 25 *μ*L mixture containing 50.0 mM KCl, 20.0 mM Tris-HCl (pH 8.8), 10.0 mM (NH_4_)_2_SO_4_, 8.0 mM MgSO_4_, 0.1% Tween 80, 0.2 *μ*M each F3 and B3, 1.6 *μ*M each FIP and BIP, 0.8 mM Betaine (Sigma), 8 U *Bst* DNA polymerase large fragment (New England Biolabs), 1.4 mM dNTPs, and a specified amount of double-stranded target DNA. The mixture was incubated at 65°C for 60 min, followed by heating at 80°C for 2 min to terminate the reaction. Products were kept at 4°C. 

### 2.4. Optimization of the Concentration Ratio between Inner and Outer Primers

In order to determine the optimal concentration ratio between inner and outer primers, 4 : 1, 8 : 1, and 10 : 1 of the concentration ratio were set in 25 *μ*L LAMP reactions by adjusting the final concentration of inner primers FIP and BIP, while the final concentration of outer primers F3 and B3 and other components remained constant. 100.0 ng/*μ*L, 50.0 ng/*μ*L, and 20.0 ng/*μ*L of the diluted *Lxx*-pMD18-T recombinant plasmid were used in 25 *μ*L LAMP reactions, of which the final concentration was 4.0 ng/*μ*L, 2.0 ng/*μ*L, and 0.8 ng/*μ*L, respectively. Three replicates were conducted for this experiment.

### 2.5. Optimization of Mg^2+^ Concentration

Mg^2+^ concentration was optimized using the condition determined to be optimal in the previous section. Nine different Mg^2+^ concentrations were tested in the 25 *μ*L of reaction system, 4.00 mM, 4.25 mM, 4.50 mM, 4.75 mM, 5.00 mM, 5.25 mM, 5.50 mM, 5.75 mM, and 6.00 mM, while the concentration of other components remained constant. 20.0 ng/*μ*L (0.8 ng/*μ*L of the final concentration in 25 *μ*L LAMP reactions) of the negative DNA extracted from *Lxx*-free juice, the positive DNA extracted from *Lxx*-infected juice, and the positive recombinant plasmid extracted from *Lxx*-pMD18-T were used in 25 *μ*L LAMP reactions, respectively.

### 2.6. Sensitivity Comparison between LAMP and PCR

Total genomic DNA was extracted from *Lxx*-infected juice and *Lxx*-infected leaf midrib of Yue Gan 18 in the mature period, respectively, with the modified CTAB extraction method which is simple, quick, and suitable for field work [20]. The quality of the DNA obtained from all these samples was assessed by agarose gel electrophoresis and the ratio of absorbance at 260 nm and 280 nm. For the juice, the third internode counted from the soil surface was used, and the juice was extracted by pressure under a sterile condition. For leaf midrib, top visible dewlap leaf midrid was ground in liquid nitrogen. Each initial concentration was 838 ng/*μ*L of the total DNA from* Lxx*-infected internode juice and 253 ng/*μ*L of the total DNA from leaf midrib. Tenfold dilutions from 10^0^ to 10^−6^ were prepared from total DNA extracted from *Lxx*-infected internode juice and leaf midrib, respectively. 1.0 *μ*L of the DNA preparations was used in both LAMP and PCR.

The PCR conditions were as follows. The primers used in PCR detection were Lxx1: 5′-CCGAAGTGAGCAGATTGACC-3′, and Lxx2: 5′-ACCCTGTGTTGTTTTCAACG-3′ [10]. The PCR reaction was carried out in a 25 *μ*L volume, including 2.5 *μ*L 10 × Ex Taq Buffer (Mg^2+^Plus) (TaKaRa Biotechnology Co., Ltd., Dalian, China), 2.5 *μ*L BSA (1.0%), 0.005 mM each dNTP, 0.005 *μ*M each primer, 0.625 U Ex-Taq DNA polymerase (TaKaRa Biotechnology Co., Ltd., Dalian, China), and 1.0 *μ*L template DNA. The conventional PCR was performed in thermal cycler (Mastercycler Gradient 96, Eppendorf, Germany) according to the following program: an initial denaturation at 95°C for 10 min, 35 cycles of denaturation at 95°C for 30 s, annealing at 56°C for 30 s, extension at 72°C for 40 s, and a final extension at 72°C for 5 min.

Additionally, sterile distilled water, 20 ng/*μ*L of total DNA extracted from *Lxx*-free internode juice, and 20 ng/*μ*L of the *Lxx*-pMD18-T recombinant plasmid were used as a blank control, a negative control, and a positive control, respectively, in the sensitivity comparison test between LAMP and PCR detection methods.

### 2.7. Analysis of LAMP Products

Stained with SYBR Green I, amplified product was detected by color change [15]. Samples that turned yellowish green were considered to be positive, while those samples that remained orange were assumed to be negative [21]. In addition, all LAMP and PCR products with an aliquot of 5 *μ*L were electrophoresed in a 2% agarose/Synergel binary gel containing ethidium bromide (0.5 *μ*g/mL) and visualized under UV light. The Presence of ladder-like DNA amplification product was considered positive reaction, while lane with no product was considered negative reaction [13].

## 3. Results and Analysis

### 3.1. Optimization of LAMP

**Figure 2:**
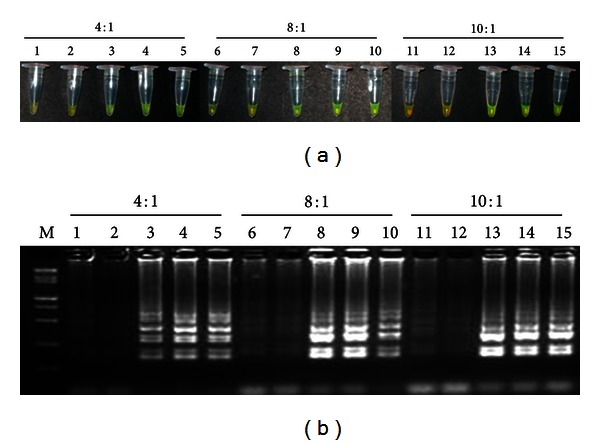
Products amplified by LAMP reactions with different concentration ratios between inner and outer primers. (a) Detection of LAMP products by color change. Samples turning yellowish green were considered positive, while the ones remaining orange were considered negative. (b) Agarose gel electrophoresis visualization of the LAMP products. Lane M, 15000 + 2000 bp marker; lanes 1, 6, and 11: the sterilized ddH_2_O; lanes 2, 7, and 12: the negative DNA extracted from the internode juice without* Lxx*; lanes 3, 8, and 13: 100 ng/*μ*L positive plasmid; lanes 4, 9, and 14: 50 ng/*μ*L positive plasmid; lanes 5, 10, and 15: 20 ng/*μ*L positive plasmid; lanes 1–5, the concentration ratio of 4 : 1 between inner and outer primers; lanes 6–10, the concentration ratio of 8 : 1 between inner and outer primers; lanes 11–15, the concentration ratio of 10 : 1 between inner and outer primers.

**Figure 3:**
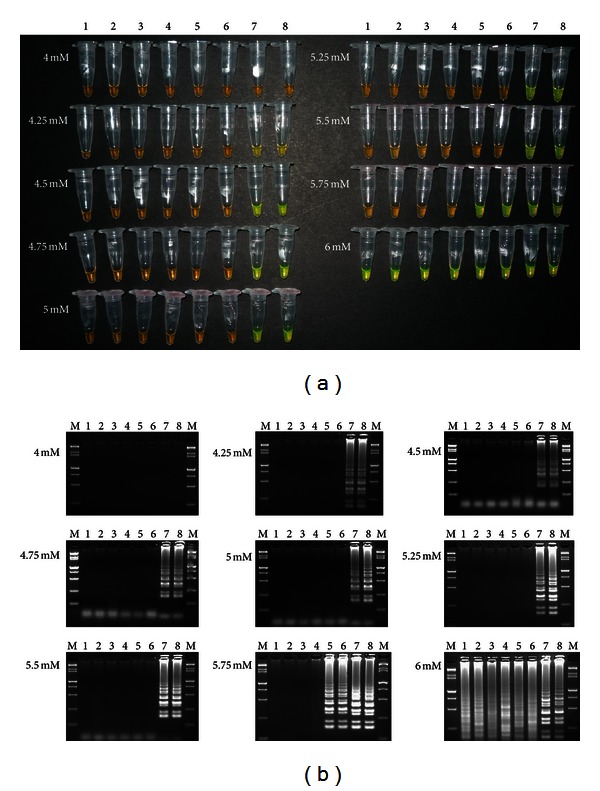
Products amplified by LAMP reactions with different Mg^2+^ concentrations. (a) Detection of LAMP products by color change. Samples turning yellowish green were considered positive, while the ones remaining orange were considered negative. (b) Agarose gel electrophoresis visualization of the LAMP products. Lane M, 15000 + 2000 bp marker; lanes 1, 2: the sterilized ddH_2_O; lanes 3, 4: 20 ng/*μ*L negative DNA extracted from the internode juice without* Lxx*; lanes 5, 6: 20 ng/*μ*L positive DNA extracted from the *Lxx*-infected juice; lanes 7, 8: 20 ng/*μ*L positive plasmid.

The effect of the concentration ratio between inner primers and outer primers and Mg^2+^ concentration on the LAMP method are shown in Figures 2 and 3. Under the 4 : 1, 8 : 1, and 10 : 1 ratios of inner and outer primers in LAMP, the tubes containing target DNA (with the target gene) turned yellowish green, while the tubes without target DNA remained orange. However, the negative color response (orange) was most obvious at the concentration ratio of 10 : 1 (Figure 2(a) tubes 11 and 12). Results obtained by agarose gel electrophoresis were similar except that there were more intense ladder-like bands at the concentration ratios of 8 : 1 and 10 : 1 (Figure 2(b)). From above, we concluded that all three concentration ratios of 4 : 1, 8 : 1, and 10 : 1 should be suitable for the follow-up optimization experiment of Mg^2+^ concentration. From the cost point of view, ratio 4 : 1 between inner and outer primers is more rational than the concentration ratios of 8 : 1 and 10 : 1, and this concentration ratio was used in the following optimization of Mg^2+^ concentration.

As showed in Figure 3(a), a concentration of 4.00 mM (Mg^2+^) in the LAMP buffer failed to produce any visible color change. When Mg^2+^ concentration increased from 4.25 mM to 5.50 mM, only the tubes with the positive plasmid *Lxx*-pMD18-T turned yellowish green. At Mg^2+^ concentrations of 5.75 mM, both the tubes with target DNA extracted from *Lxx*-infected juice and the plasmid DNA of *Lxx*-pMD18-T turned yellowish green. When the concentration of Mg^2+^ increased to 6.00 mM, all tested samples turned yellowish green even with the blank control (sterile distilled water) and the negative control. Three independent experiments produced the same results. Similar results were also observed in the detection by agarose gel electrophoresis (Figure 3(b)). 

### 3.2. Optimized LAMP Method for the Detection of *Lxx*

Based on the optimized reaction conditions described above, the LAMP assays for detecting *Lxx *in sugarcane was established. The LAMP reaction used in further experimentation was carried out in a 25 *μ*L reaction mixture system containing 10 mM KCl, 20 mM Tris-HCl (pH 8.8), 10 mM (NH_4_)_2_SO_4_, 5.75 mM MgSO_4_, 0.1% Triton X-100, 0.2 *μ*M each F3 and B3, 0.8 *μ*M each FIP and BIP, 8 U* Bst *DNA polymerase large fragment (New England Biolabs), and 1.4 mM dNTPs.

### 3.3. Sensitivity Comparison between LAMP and PCR

**Figure 4:**
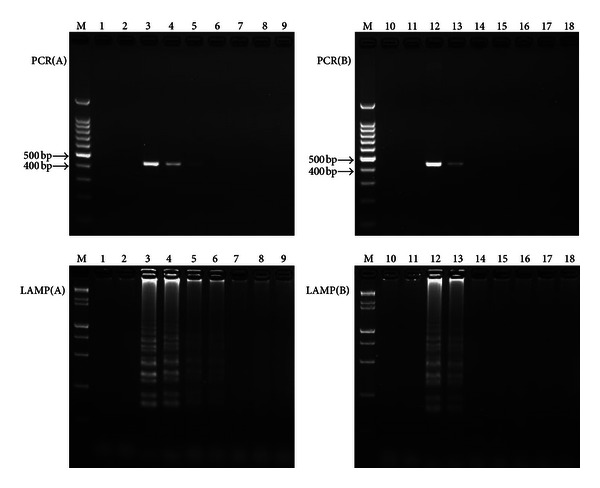
Agarose gel electrophoresis visualization of PCR and LAMP products. PCR(A) and PCR(B), Lane M is 100 bp marker; LAMP(A) and LAMP(B), Lane M is 15000 + 2000 bp  marker. Lanes 1, 10: the sterilized ddH_2_O; lane 2, the negative DNA extracted from the internode juice without* Lxx*; lane 11, the negative DNA extracted from the leaf midrib without* Lxx*; lanes 3–9, the amplification products of 10^0^–10^−6^ concentration gradient of positive DNA extracted from the *Lxx*-infected internode juice; lanes 12–18, the amplification products of 10^0^–10^−6^ concentration gradient of positive DNA extracted from the *Lxx*-infected leaf midrib.

The initial concentration of the total DNA extracted from the internode juice and the leaf midrib in 25 *μ*L reaction mixtures were 33.52 ng/*μ*L and 10.12 ng/*μ*L, respectively, for both the LAMP and PCR protocols. Within the dilution series of the total DNA extracted from *Lxx*-infected internode juice, the 10^−2^ dilution was the lowest concentration in which *Lxx *was detected by PCR, while *Lxx* could be detected by LAMP in the 10^−3^ dilution concentration. The 10^−1^ dilution was the lowest concentration of DNA extracted from* Lxx*-infected leaf midrib in which *Lxx *was detected by PCR and LAMP (Figure 4). 

Therefore, for PCR detection, the sensitivity for the total DNA extracted from internode juice and that from leaf midrib as templates were 0.3352 ng/*μ*L and 1.012 ng/*μ*L, respectively. For LAMP reactions, the sensitivity with the total DNA extracted from internode juice and that from leaf midrib were 0.03352 ng/*μ*L and 1.012 ng/*μ*L, respectively. It can be concluded from these results that when total DNA extracted from internode juice was used as the template, the sensitivity of LAMP was 10 times higher than that of the conventional PCR detection.

## 4. Discussion

LAMP, a novel nucleic acid amplification method, is a promising new technique [13]. In the present study, we developed an LAMP assay for *Lxx*. This is a simple and feasible diagnostic tool in which the reaction takes place in a single tube incubated in a heat block for 62 min compared to conventional PCR that takes about 2 h to detect *Lxx* and requires expensive and specialized equipment such as a thermal cycler and a gel scanner [10]. The adoption of a pathogen-free sugarcane seed cane program in sugarcane planting countries including China requires a rapid, simple, and sensitive detection of *Lxx*. To our knowledge, the LAMP-based assay for *Lxx* developed in this study is the first of its kind for pathogen detection in sugarcane.

Our study supports the view of the original developer of the LAMP technique [13] and subsequent researchers that the LAMP assay is useful for rapid detection and diagnosis because it can be efficiently performed with limited resources and has the potential to be used in field condition [16, 18, 22]. The amplification efficiency of the LAMP method is extremely high because all reactions are conducted at constant, optimal temperature suitable for the enzyme, no time-consuming thermal changes used in conventional PCR are required, and visual evaluation of the reaction mixture can be made immediately without the added step of gel electrophoresis required for conventional PCR analysis. Though the steps are simple, the efficiency, sensitivity, and quantitative capability of LAMP reaction strongly depend on primer design. The LAMP reaction requires four sets of primers targeting six distinct target regions, making primer design more complex and difficult than that in conventional PCR. What should also be stressed is that in the present study, in order to prevent false positive amplification caused by aerosol with the opening of PCR tube lid to add the SYBR Green I, one drop of SYBR Green I was placed right at the tube lid in advance before it is being covered. Once the LAMP reaction is finished, the preadded SYBR Green I at the tube lid can be centrifuged into the reaction mixture to trigger color reaction and thus the color change [15]. 

The optimization of reaction system is necessary for sensitive and specific detection. Several parameters, including the concentration ratio between inner and outer primers, dNTPs concentration, and Mg^2+^ concentration, play a significant role in LAMP [15, 18, 23]. Guan et al. [15] found the optimization of different primer concentrations and the ratios between inner primers (FIP and BIP) and outer primers (F3 and B3) to be essential for the development of a LAMP assay to detect genetically modified soybean events. In this study, as shown in Figure 2, we can conclude that among the three concentration ratios of 4 : 1, 8 : 1, and 10 : 1, 4 : 1 between inner and outer primers is more rational than the concentration ratios of 8 : 1 and 10 : 1, and this concentration ratio was used in the follow-up optimization experiment of Mg^2+^concentration. Previous studies revealed that, since Taq polymerase is a magnesium-dependent enzyme, the optimal concentration of Mg^2+^ is critical to the success of the PCR reaction [24]. They also found that primers which bind to incorrect template sites are stabilized in the presence of excessive magnesium concentrations and thus results in decreased specificity of the reaction, while excessive magnesium concentrations may also stabilize double stranded DNA and prevent complete denaturation of the DNA during PCR and so reduces the product yield [24]. On the other hand, inadequate MgCl_2_ may result in the formation of concentration gradients within the magnesium chloride solution supplied with the DNA polymerase and also results in many failed experiments [24]. Nie [18] made a similar observation on Mg^2+^concentration when developing an RT-LAMP assay for detection of Potato virus Y. Although the critical value of the Mg^2+^ concentration differed, another researcher found the Mg^2+^ concentration to be the most critical component in optimizing their LAMP assays [14–16, 18]. In our study, the Mg^2+^ concentration was proved to be the most crucial factor affecting the sensitivity of *Lxx*-LAMP assay and the optimal Mg^2+^ concentration is 5.75 mM, while false negative results are obtained at the concentration from 4.25 mM to 5.50 mM and false positive results at the concentration of 6.00 mM.

Under the above conditions, the *Lxx-*LAMP protocol was capable of detecting a 10^−2^ dilution of the total DNA extracted from *Lxx*-infected internode juice, a 10-fold higher level of sensitivity than that of the PCR method. Similar sensitive levels were obtained in the other LAMP assay system [15]. And Kaneko et al. [25] found that the sensitivity of LAMP was less affected by the various components of the DNA samples than was PCR. According to the results of sensitivity comparison between LAMP and PCR method, when the total genomic DNA extracted from internode juice was used as the template, 0.3352 ng/*μ*L and 0.03352 ng/*μ*L can be detected by PCR and LAMP, respectively. From above, when total DNA extracted from internode juice was used as the template, the sensitivity of LAMP was 10 times higher than that of the conventional PCR detection, while when the total genomic DNA extracted from leaf midrib was used as the template, both PCR and LAMP can detect the genomic DNA at the same lowest concentration of 1.012 ng/*μ*L. It indicated that the concentration of the target pathogen of *Lxx *in internode juice was higher than that in leaf midrib. It may be because there were smaller, less mature vascular tissues in the leaf midrib. Besides, we can conclude that the LAMP is more sensitive than the conventional PCR to detect *Lxx* in the internode juice.

## 5. Conclusions 

A visual and rapid detection method for xylem-limited coryneform bacterium *Lxx* was developed. This is a simple, feasible, and time-saving diagnostic tool in which the reaction takes place in a single tube incubated in a heat block for 62 min compared to conventional PCR that takes about 2 h to detect *Lxx* and without the need for expensive and specialized equipment such as a thermal cycler and a gel scanner [10]. To our knowledge, the LAMP-based assay for *Lxx* developed in this study is the first of its kind for pathogen detection in sugarcane. In the present study, positive and negative samples detected by the LAMP method were clearly distinguishable. In addition to the application of LAMP for the detection of *Lxx*, we believed that the LAMP assays can also be developed and applied to the detection of other pathogens in sugarcane. Also, there may be a potential use of LAMP for detecting GMOs in sugarcane.

## Figures and Tables

**Table 1:** Sequences of LAMP primers used for detection of *Lxx*. The “c” indicates the reverse and complementation of the “F1” or “B2” sequence shown in Figure 1.

Primer	Sequence (5′-3′)
F3	ACATCGGTACGACTGGGT
B3	TGGCCGACCAAAAAAGGT
FIP (F1c + F2)	GGCGTACTAAGTTCGAGCCGTT-GGTCAGCTCATGGGTGGA
BIP (B1 + B2c)	CCTCGCACATGCACGCTGTT-CTCAGCGTCTTGAAGACACA
